# A Case of Cauda Equina Injury Through an Anterior Abdominal Stab Wound

**DOI:** 10.5435/JAAOSGlobal-D-22-00106

**Published:** 2023-01-11

**Authors:** Amelia V. Suddaby, David Yen

**Affiliations:** Department of Orthopaedic Surgery, Queen's University, Kingston, Canada.

## Abstract

This case study discusses a unique case of a cauda equina injury through an anterior abdominal stab wound that was missed on initial assessment in the trauma bay. The true nature of the injury was only discovered intraoperatively, despite preoperative clinical findings and imaging. A literature review was conducted, and only one other instance of anterior stab injury to the spine was reported. The purpose of this case study was to allow for earlier recognition of anterior transection injuries of the cauda equina and to highlight the importance of advanced imaging and exploratory surgery in cases of abdominal penetrating injuries presenting in conjunction with neurologic deficits.

This case study discusses a unique case of cauda equina injury through an anterior abdominal stab wound. This injury was missed on initial assessment, and the true nature of the injury was discovered intraoperatively. The purpose of this case study was to allow for earlier recognition and treatment of anterior transection injuries of the cauda equina and/or spinal cord. Approval was obtained by the committee on research ethics, and informed consent was obtained from the patient.

A literature review of EMBASE and MEDLINE was done. Several posterior stab injuries to the spine were reported, but only one instance of anterior stab injury to the spine was described in a 2012 case study.^1^

## Case Description

This is a case of a 31-year-old man who was brought to the emergency department after sustaining stab wounds to the neck and abdomen with a filet knife. The initial trauma notes by the trauma team physicians described the abdominal wound as being just above the umbilicus, approximately 2 cm in width and with approximately 8 to 10 cm of omentum protruding from the wound. On arrival to the emergency department, the patient was hemodynamically stable with a Glasgow Coma Scale score of 15 and had a benign abdominal examination and negative focused assessment with sonography in trauma examination. On neurological examination, which was completed according to the Asia Spinal Injury Association scoring, he was found to have 1/2 sensation over L4 and 0/2 sensation to the L5 and S1 distributions in the right lower extremity and 0/5 ankle dorsiflexion, ankle plantar flexion, and great-toe extension on the right side. Right-sided hip flexion and knee extension were 4/5, and sensation in the L2-L3 distributions was 2/2. These deficits were reported as being new by the patient, but were felt not to be in keeping with his mechanism of injury. He was also found to have slightly decreased rectal tone on digital rectal examination. Given the patient's active intravenous drug use intravenous drug use, current foot cellulitis, and recent knee effusion, there was some thought that his motor examination findings might be more in keeping with an epidural abscess than as a sequela of his trauma, and therefore, this was on the list of differential diagnoses.

**Figure 1:**
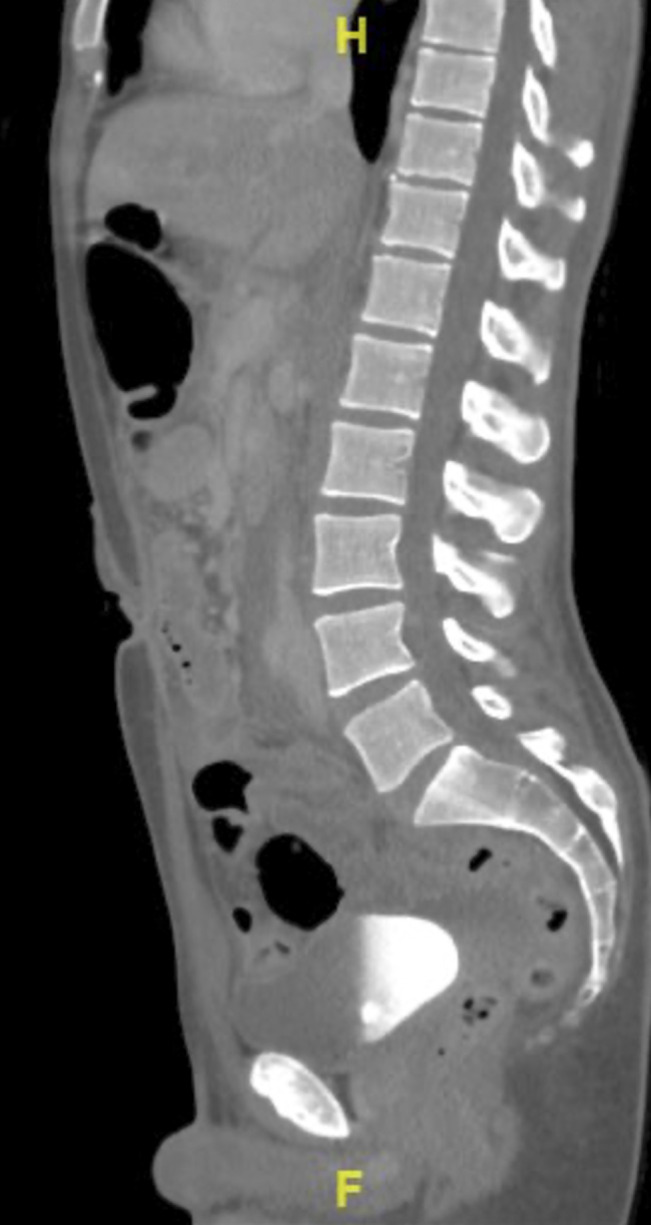
Sagittal and axial CT cuts showing a 2.4 × 8.6 cm retroperitoneal hematoma but no abnormality in the spine at L3-4.

**Figure 2:**
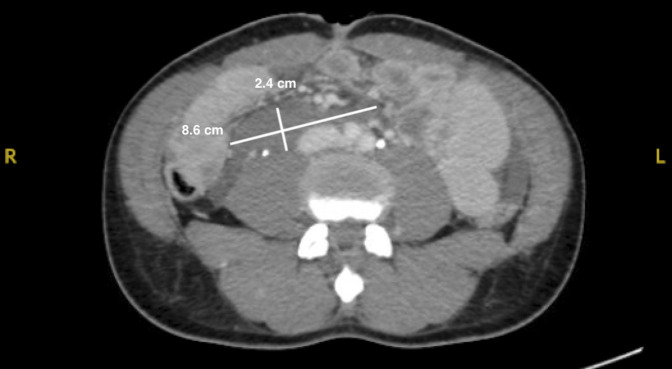
Sagittal and axial CT cuts showing a 2.4 × 8.6 cm retroperitoneal hematoma but no abnormality in the spine at L3-4.

The patient was transported to the CT scanner before exploratory laparotomy. The CT scan was reported as showing the aforementioned abdominal wound and moderate-volume retroperitoneal hemorrhage, small-volume hemoperitoneum, and possible distal ureteric injury (Figures 1 and 2).

During exploratory laparotomy conducted that same evening, a proximal through-and-through jejunal injury was identified and repaired primarily. Blood and clot were found within the abdomen, but there was no laceration noted to the posterior peritoneum.

**Figure 3:**
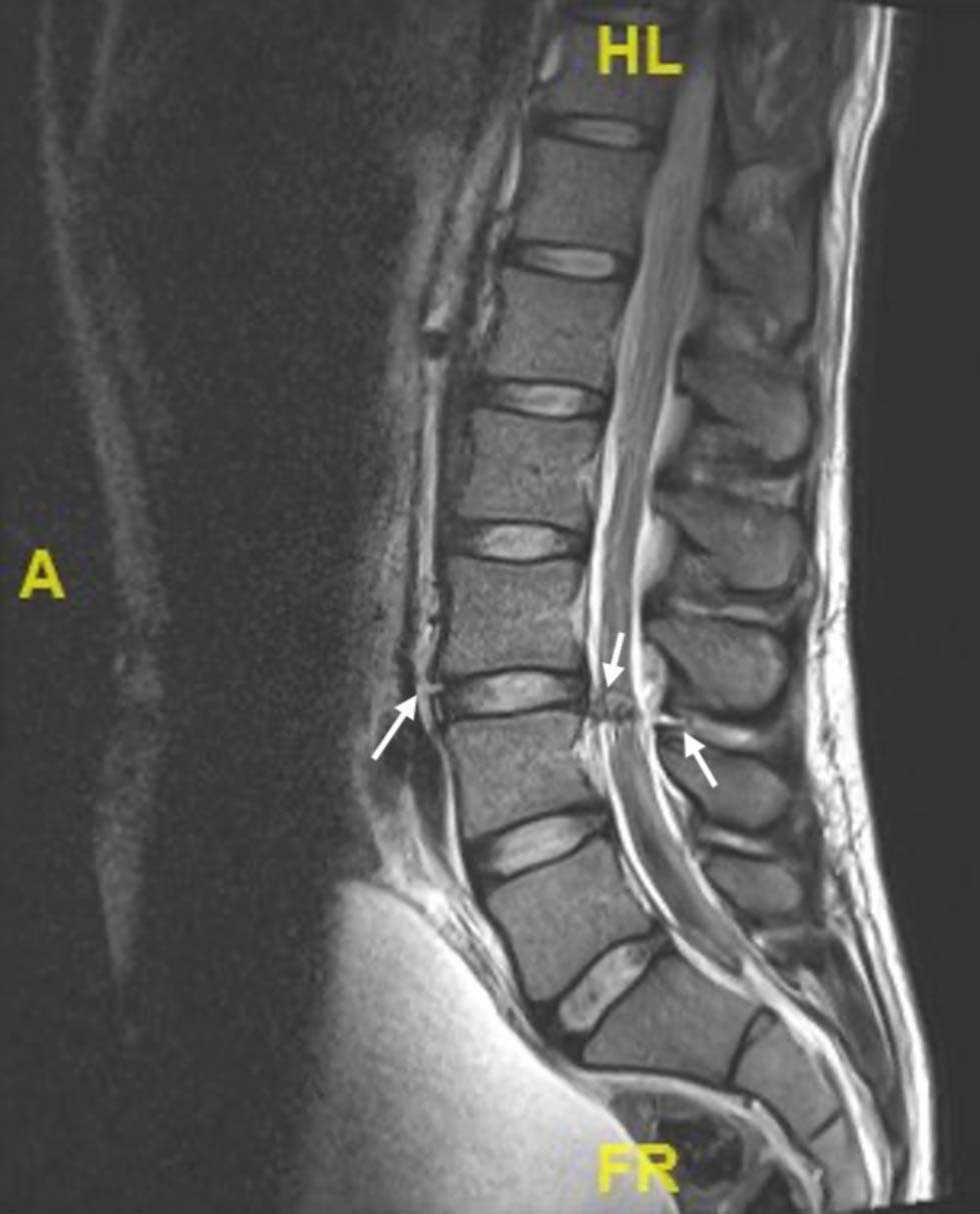
Sagittal MRI cut showing reported disk extrusion. Arrows demonstrate disruption of the anterior longitudinal ligament.

**Figure 4:**
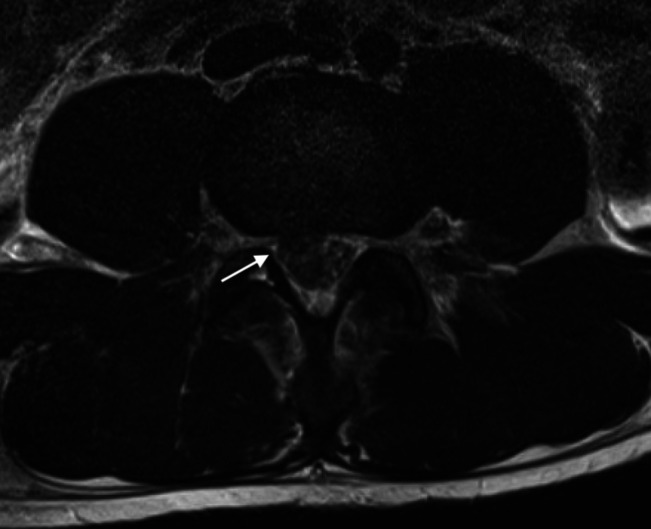
Axial MRI cut at L3-4 showing reported disk extrusion.

Postoperatively, the patient had difficulty voiding and required a number of straight catheterizations before indwelling Foley catheter insertion. Given the patient's neurologic symptoms, MRI of the spine was done on the day after admission and was reported as showing a focal central/right subarticular disk extrusion at the level of the L3-L4 disk space, with approximately 10-mm disk extension into the spinal canal, abutting and likely compressing the traversing nerve roots at this level (Figures 3 and 4). Contrast was not used, and the MRI was reported by a neuroradiologist. No mention was made of any disruption of the anterior longitudinal ligament, ligamentum flavum, or posterior longitudinal ligament.

Although we recognized that the patient's neurological deficits did not correspond with the findings reported on the MRI scan, we thought that surgical exploration and decompression was warranted given the patient's ongoing neurological symptoms. He was brought to the operating room two days after admission for a posterior L3-L4 decompression. As previously reported, the patient had no sensation in this foot and was unable to dorsiflex or plantarflex his ankle or extend his great toe. Hip flexion and knee extension were intact.

**Figure 5:**
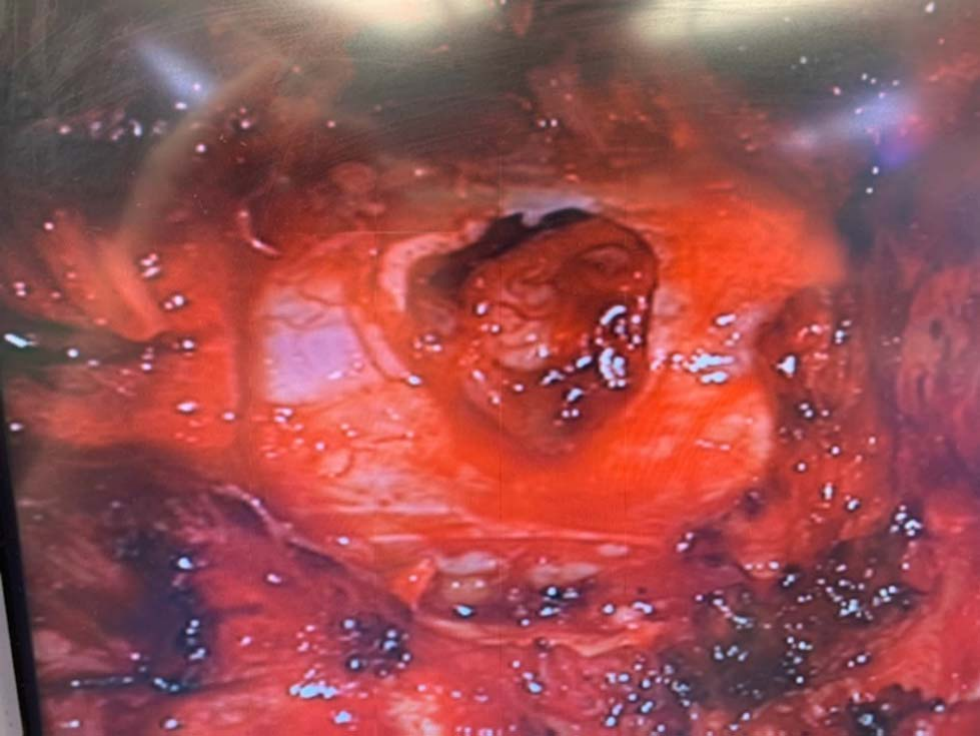
Intraoperative image showing dorsal dural laceration.

**Figure 6:**
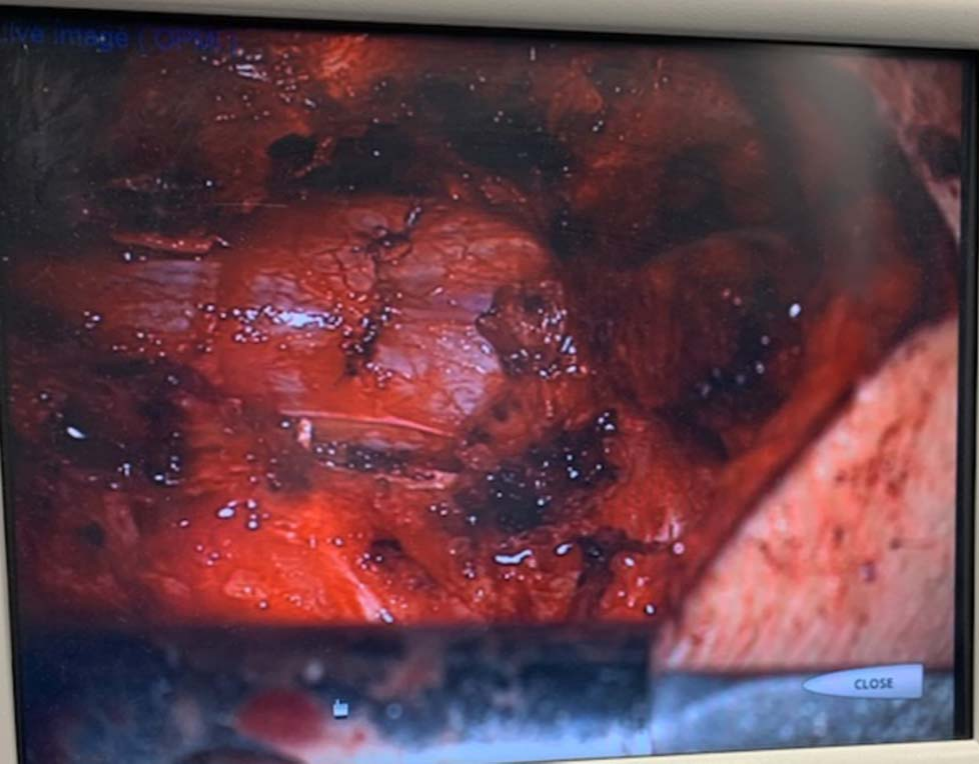
Intraoperative image after primary repair of dorsal dural laceration.

Intraoperatively, posterior decompression was conducted, revealing disruption of ligamentum flavum and the presence of cerebrospinal fluid (CSF) and transected cauda equina nerve rootlets. Additional exposure of the dura revealed a sharp horizontal laceration of its dorsal aspect at the L3-L4 level, with extrusion of transected right-sided nerve rootlets (Figure 5). These were reduced back into the thecal sac, and primary repair of the posterior dural laceration was done using a running stitch of 5 to 0 silk suture (Figure 6). Anterior dural exploration was deferred by the operating surgeon because there was no CSF coming from anteriorly, and it was thought that exploring the anterior dura might lead to anterior CSF extravasation. Ultrasonography was used to confirm that there was no external impingement on the thecal sac, and a Valsalva maneuver was used to confirm that there was no CSF leak after the repair. Tisseel was then applied over the exposed dura, and sterile absorbable gelatin sponge was placed over the Tisseel to fill in the potential space left by the decompression. No disk herniation was noted intraoperatively. No specific restrictions were placed regarding mobilization postoperatively.

Postoperatively, the patient continued to have decreased sensation to the S1 dermatome and absent motor function to L4-S1. His Foley catheter was removed on postoperative day two, and he was voiding well. He was placed in a foot drop brace.

**Figure 7:**
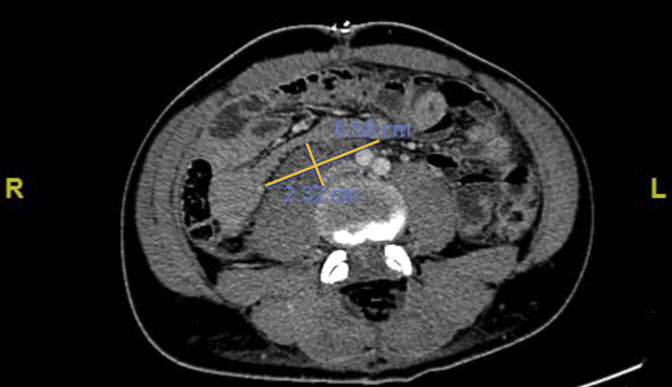
Axial CT cuts showing a smaller (2.3 × 6.6 cm) retroperitoneal hematoma.

Additional imaging was conducted 7 days after admission to ensure no additional retroperitoneal bleeding was present. This showed residual lower abdominal retroperitoneal hematoma with mass effect on the inferior vena cava, which had decreased in size compared with prior imaging. No definite vascular injury was identified (Figure 7).

The patient was seen at the clinic six and a half months after his surgery. He continued to have absent motor function in ankle dorsiflexion, plantar flexion, and great-toe extension. He also had decreased sensation to light touch from the midcalf down. He continued to wear his ankle-foot orthosis and endorsed radicular type pain to his right leg, which responded to pregabalin.

## Discussion

In summary, this case report highlights an instance of missed penetrating, transecting injury to the cauda equina from an anterior stab wound to the abdomen, with the full extent of injuries not being revealed until the time of surgery. A review of literature shows that this is a unique case of anterior-penetrating injury to the cauda equina. As mentioned previously, only one instance of anterior stab injury to the spine was described in a 2012 case study,^1^ involving retention of a piece of glass that was removed six weeks after the initial injury. In this case report, a 21-year-old man sustained a single stab injury to the abdomen with a broken glass beer bottle. Emergency exploratory laparotomy was conducted, and advanced imaging of his abdomen and spine was, therefore, never completed during his hospital admission. His postoperative concerns of ongoing pain and mild limp were attributed to his abdominal surgery and notable hollow viscus injuries. He was referred to the orthopaedic team 1 month after discharge from hospital with severe sciatica to his left leg. The authors of this case report concluded that CT of the spine should be done on all patients with penetrating trauma to the spine, in which routine radiographic evaluation was deemed to be inadequate. They also stated that indications for surgical management of spinal stab wounds were rare but elected to conduct surgery on their patient given the persistent, severe sciatica, which was still present one month after discharge from hospital.

A CT scan was not helpful in our case because neither fracture nor retained foreign bodies were present, and no spinal abnormality was found. It was, however, useful in identifying a retroperitoneal hematoma, which alerted us to potential injury to the great vessels. MRI was used to identify our spinal injury. Although the radiographic abnormalities were initially interpreted as representing a disk extrusion, a retrospective review of the preoperative MRI does reveal a laceration of the anterior longitudinal ligament and ligamentum flavum on the T2-weighted sagittal MRI and laceration of the posterior longitudinal ligament on the T1-weighted sagittal MRI, which could only occur with associated-penetrating trauma to the cauda equina. With this experience, we would recommend that an MRI, rather than (or in addition to) a CT scan, should be done in the presence of neurologic deficit in cases of anterior abdominal penetrating injuries.

We found transected and extruded nerve rootlets at the time of surgery which, if left untreated, could result in intractable radicular or dysesthetic pain. Therefore, in the presence of MRI abnormalities in cases of anterior abdominal penetrating injuries, we would recommend early exploratory surgery and appropriate surgical management.

## Conclusion

It is our hope that in presenting this case report, we will bring awareness to the possibility of anterior penetrating trauma to the cauda equina in the case of abdominal stab wounds. We think that careful neurological examination should be conducted in any penetrating abdominal trauma and, if abnormal, an MRI be done. If the MRI is suspicious for a spinal injury, we recommend early surgical intervention.
